# Effects of deliberate reflection on diagnostic accuracy, confidence and diagnostic calibration in dermatology

**DOI:** 10.1007/s40037-019-0522-5

**Published:** 2019-07-09

**Authors:** Galileu B. Costa Filho, Alexandre S. Moura, Paulo R. Brandão, Henk G. Schmidt, Silvia Mamede

**Affiliations:** 1 Universidade José do Rosário Vellano, Belo Horizonte, Brazil; 2 Institute of Medical Education Research Rotterdam, Erasmus MC, University Medical Center Rotterdam, and Department of Psychology, Erasmus University Rotterdam, Rotterdam, The Netherlands

**Keywords:** Diagnostic calibration, Deliberate reflection, Clinical reasoning, Medical education

## Abstract

**Background:**

Deliberate reflection on initial diagnoses improved diagnostic accuracy in internal medicine and general practice, but it is unknown if the same occurs in specialties that rely mostly on visual perception, such as dermatology. Moreover, whether reflection influences diagnostic calibration has not been studied yet. Diagnostic calibration, the relationship between diagnostic accuracy and confidence in that accuracy, affects diagnostic performance because overconfidence tends to induce premature closure. This study evaluated the effects of deliberate reflection on diagnostic accuracy and diagnostic calibration in dermatology.

**Methods:**

Sixty-one sixth-year students from a Brazilian medical school were allocated to either a reflection group (RG) or a control group (CG). In both groups, students worked with the same 12 dermatological images, presented sequentially, providing an initial diagnosis and confidence in that diagnosis. Subsequently, RG students reflected on the case using a structured procedure, while CG students performed a time-filler activity. All students then provided a final diagnosis and confidence in that diagnosis. Outcome measurements were diagnostic accuracy, confidence, and calibration.

**Results:**

Reflection increased diagnostic accuracy relative to control (49.7 ± 12.1 vs 38.4 ± 14.6; *p* = 0.002) but did not affect confidence (64.3 ± 13.2 vs 58.9 ± 20.1; *p* = 0.228) nor calibration (0.15 ± 0.16 vs 0.20 ± 0.19, *p* = 0.197). Overall, case difficulty influenced calibration, with students showing more overconfidence on more difficult cases (*p* <0.001).

**Conclusions:**

Deliberate reflection increased diagnostic accuracy in dermatology but did not affect confidence and calibration. Calibration was worse on more difficult cases, suggesting that calibration is a knowledge-related phenomenon.

## What this paper adds

Overconfidence is one of the most common biases in diagnostic reasoning, adversely affecting physicians’ decision-making, but there is little empirical research on strategies to improve diagnostic calibration. This experiment assessed the effect of deliberate reflection on diagnostic accuracy, confidence and calibration of dermatological lesions. We have shown that deliberate reflection improved diagnostic accuracy but did not improve calibration, particularly due to its minimal effect on the student confidence in their diagnostic accuracy.

## Introduction

Medical error has been recognized as an important cause of adverse effects and deaths since the highly cited 1999 Institute of Medicine report called attention to the problem [1]. A recent review estimated that up to 400,000 deaths a year could be attributed to medical error [2]. Diagnostic errors have been acknowledged as one of the most common and harmful problems affecting patient safety and are the leading cause of paid malpractice claims, with higher rates in specialties that deal with high degrees of clinical uncertainty [3–5].

Several factors usually underlie an error, but flaws in physicians’ reasoning play a key role. Faulty reasoning contributed to around 75% of the diagnostic errors investigated in US academic hospitals and is also highly prevalent in outpatient care [6, 7]. These reasoning flaws have been associated with excessive reliance on first diagnostic impressions generated through intuitive judgments [8]. In routine situations, physicians generate diagnostic hypotheses through a fast, to a large extent automatic, ‘matching’ of the case at hand with examples of previous patients or prototypes of diseases stored in memory [9]. This process of ‘pattern recognition’ is usually efficient, but when initial hypotheses are wrong, they can only be corrected if a more thorough analysis of the case takes place [8, 10]. If the physician then ‘trusts’ too much his/her initial impression, does not submit it to a more careful analysis and consider alternative hypotheses, an error would occur. When this happens, the physician is usually said to have fallen into ‘premature closure’, which has been shown to be a major source of cognitive diagnostic errors [6].

Premature closure may be more likely to occur when there is a mismatch between the physician’s confidence in the diagnosis and his/her actual performance. This seems reasonable to expect, because a clinician who is highly confident about having established a correct diagnostic hypothesis would tend to be less motivated to scrutinize it. When his/her confidence is unjustified because the diagnostic hypothesis is in fact wrong, the physician is said to be, in this specific situation, overconfident [11]. Overconfidence reflects a poor calibration between the professional’s confidence in the diagnosis he/she made for a particular case and the accuracy of such diagnosis [11]. Empirical evidence, though scarce, suggests that physicians may have a tendency to underestimate the likelihood that their diagnoses are incorrect [12, 13]. Overconfidence has indeed been described in psychology research as a robust phenomenon linked to biases in information processing such as a tendency to selectively focus on evidence that supports one’s initial impressions [14, 15].In medicine, overconfidence has been said to open the door for reasoning flaws such as confirmation bias and premature closure adversely affecting physicians’ decision-making [11, 16, 17]. Together with other sources of cognitive error such as availability and anchoring heuristics, and personality traits such as lower tolerance to risk, they seem to play a major role in diagnostic inaccuracies [16].

Much attention has been drawn to strategies that can possibly help prevent such biases and reduce diagnostic errors derived from reasoning flaws [18]. One of these strategies is deliberate reflection on clinical cases, which several studies have shown to improve diagnostic accuracy [19, 20] and counteract cognitive biases such as the availability bias [21, 22].

In these studies, deliberate reflection required physicians to go back to the case that they had just diagnosed and look for evidence that contradicts their initial diagnostic hypothesis, generating and contrasting alternative diagnoses. By doing so, physicians can recognize relevant clinical findings that have gone initially unnoticed, which may also be helpful to improve diagnostic calibration. Indeed, this expectation seems in line with research in psychology showing that judgments were better calibrated, especially regarding overconfidence, when people were requested to search for evidence that contradicts their first impressions [14].

However, previous studies that have requested physicians or medical students to deliberately reflect were not concerned with diagnostic calibration but only with diagnostic accuracy. Whether deliberate reflection affects calibration remains unknown. If reflection on one’s own perception of a visual pattern works to reveal that inferences made about characteristics of the lesions are not so unquestionable, this could generate doubt and uncertainty, consequently reducing a tendency towards overconfidence. Moreover, concerning the positive effects of reflection on diagnostic accuracy, previous research has been confined to medical specialties such as internal medicine and family medicine, which require physicians to interpret and integrate a diversity of clinical findings and involve sequential judgments. It is not known whether there would be any benefit of reflection in specialties that are highly visual, such as dermatology, when diagnosis tends to be based on a holistic pattern-recognition process.

This experimental study investigated the effects of a deliberate reflection procedure adapted for the diagnosis of dermatology cases on diagnostic accuracy and diagnostic calibration, i.e., the relation between confidence in the diagnosis and accuracy. Final-year medical students were asked to diagnose a set of dermatology cases either by following the deliberate reflection procedure or their conventional approach. We hypothesized that deliberate reflection would improve diagnostic accuracy and calibration.

## Method

### Design

We conducted a single-phase quasi-experimental study. Students were systematically allocated to either a reflection group (RG) or a control group (CG). In both groups, students worked with the same 12 dermatological images, in a sequential order. In both groups, students were instructed to provide an initial diagnosis and their confidence in that diagnosis. Subsequently, while RG students reflected on the case using a structured procedure, CG students engaged in a time-filler activity. All students then provided a final diagnosis and their confidence in that diagnosis.

### Participants

Students enrolled in the sixth year of the Universidade José do Rosário Vellano (Unifenas) Medical School, Belo Horizonte, Brazil were invited to participate in this study. The medical school has a six-year problem-based learning curriculum. The students recruited from the final year of the course had already had contact with patients with dermatological lesions in the fourth year of the course and during their family medicine clerkship that is placed in the sixth year. All volunteers gave written consent to participate in the study.

Ethical approval for the study was granted by our institutional review board (Universidade José do Rosário Vellano Research Ethics Board, #1.931.044). The study was carried out in accordance with the Declaration of Helsinki.

## Materials and procedure

Before the beginning of the study, student self-perception of experience and knowledge of dermatological lesions was evaluated. A form containing 20 dermatological diagnoses (12 diagnoses used in the study and 8 distractors) was handed out to the students and, for each listed diagnosis, the student rated his/her self-perceived prior knowledge and clinical experience on a 5-point Likert scale.

Twelve images of dermatological lesions were used in this study. The diagnoses were: psoriasis, impetigo, contact dermatitis, pemphigus, hives, leprosy, ringworm, basal-cell carcinoma, pigmented seborrhoeic keratosis, melanocytic nevus, congenital nevus, and melanoma. Prior to this study, a pilot was conducted with internal medicine residents and fourth-year medical students to evaluate the level of diagnostic complexity of the images. Two lesions were excluded: a lichen planus lesion, because none of the students were able to confirm the diagnosis, and a tinea cruris lesion, because all the students provided the correct diagnosis. These lesions were substituted by a contact dermatitis lesion and a tinea corporis lesion, respectively.

The study was conducted right after regular didactic lectures with six groups of students who were, at the moment of the study, in different clinical rotations (Obstetrics, Gynaecology, Primary Care, Specialty Care, Emergency Care, Neonatal Care). In a sequential and alternate manner (i.e., the first rotation to the experimental group, the second to control group and so forth), we allocated the whole group of students enrolled in a clinical rotation to either the Reflection Group (RG) or Control Group (CG), an approach that would help to prevent contamination.

Images of the dermatological lesions were presented to the students in a notepad, where each page contained the image of one lesion with no medical history. For each image, the students from both groups were told to provide, in 30 s, an initial diagnostic hypothesis and assign their confidence in the accuracy of the diagnosis by using a scale ranging from 1 to 10 (1 equivalent to ‘I’m not confident’ and 10 to ‘I’m fully confident’).

Thereafter, the student performed a second task, lasting 3 min and 30 s, which differed depending on the experimental condition to which the student had been allocated. The RG students were instructed to engage in reflection on the case, following a procedure in which they should (1) list the visual aspects that supported their initial diagnosis and (2) those that spoke against their diagnostic hypothesis. Participants were subsequently asked to list two alternative diagnoses assuming that the initial diagnosis generated for the lesion proved to be incorrect, and to follow the same procedure (steps 1 and 2) for each alternative diagnosis. Finally, they were asked to select their final diagnosis for the case and assign their degree of confidence in diagnostic accuracy (in the same scale used to evaluate the confidence on the accuracy of the initial diagnostic hypothesis). For the CG students, the second task was a distractor activity which consisted of finding nine medical terms (not related to the lesions used in the study) in a crossword. After that, the students were then asked to provide their final diagnosis and their confidence in the accuracy of this diagnosis. At the end of the activity, the students from both groups answered a final question on the number of lesions from the study that they believed they had correctly diagnosed.

### Data analysis

Two experts (P.R.B and G.B.C) independently assessed the participants’ diagnoses blinded to the experimental conditions under which they were provided. The diagnoses were evaluated as fully correct or incorrect and scored as 1 or 0 points, respectively, and the total percentage of correct diagnoses was computed.

For confidence, the scores given on each case were summed and a percentage of the total confidence was computed. Subsequently, the mean percentage of correct diagnoses and the mean percentage of confidence in the correctness of the diagnosis were computed for each experimental condition.

Calibration was defined as the alignment between students’ confidence and accuracy. We evaluated such alignment using the ‘over-under’ (O-U) index, which indicated the direction and magnitude of calibration error, ranging from −1 (highest possible level of underestimated confidence) to +1 (highest level of overestimated confidence) [23]. The O‑U index was calculated as the difference between the percentage of correct diagnoses and the percentage of confidence divided by 100.

We used Pearson’s χ^2^ test to compare baseline categorical variable (gender) and Student’s t‑test for baseline continuous variables (age, self-perception of prior knowledge and experience) between groups. Diagnostic accuracy in the initial and final phases (initial diagnosis and diagnosis after the task), final confidence, and final calibration were compared between experimental conditions using independent t‑tests. In addition, to check whether there was a variation in accuracy between the initial and the final diagnosis which differed between the two conditions, we computed the ‘gain’ in diagnostic accuracy by subtracting the initial diagnosis from the final diagnosis and performed an independent t‑test on the ‘gain’. Effect sizes were analyzed by computing Cohen’s d.

Pearson’s correlation coefficient was used to analyze the relationship between the students’ estimation of right answers (0 to 12) and their actual diagnostic accuracy.

As no overall effect of the reflection on the diagnostic calibration was observed, a post-hoc analysis was carried out to better understand the phenomenon. A mixed analysis of variance (ANOVA) was performed with experimental conditions as a between-subjects factor (reflection group vs control group) and case difficulty as within-subjects factor (easy vs difficult) on calibration. The difficulty of the cases was defined by using the performance data of the students in the study itself. The six lesions in which the students showed the best performance (i.e. the six highest diagnostic accuracy scores) were classified as ‘easy’ and the remaining lesions as ‘difficult’.

## Results

Sixty-one of 90 invited students agreed to participate in the study and were allocated to the RG (*n* = 33) or the CG (*n* = 28). The proportion of women among the participating students was larger in the RG than in the CG (72.7% vs 42.9%; χ^2^(1) = 5.59, *p* = 0.035). No difference in age was observed when the RG and CG were compared (27.1 ± 4.3 vs 27.7 ± 5.9 years, respectively; *p* > 0.619). No samples were lost, and no students were excluded. The students from the two groups showed no statistically significant difference regarding the degrees of self-perception of prior knowledge (3.5 ± 0.8 vs 3.4 ± 0.6; *p* = 0.449) and clinical experience related to the dermatological diseases (2.9 ± 0.8 vs 2.7 ± 0.7; *p* *=* 0.277).

Table 1 presents the results for diagnostic accuracy, confidence and calibration for the two experimental conditions. Whereas diagnostic accuracy did not significantly differ between the two conditions in their initial diagnosis of the dermatological lesions, a significantly higher final diagnostic accuracy was observed in the RG relative to the CG. Indeed, the analysis of the difference between initial and final diagnostic accuracy scores (i.e. before and after the given task) shows that the two conditions significantly differ in the gain across the two diagnoses [RG and CG, mean difference (standard deviation), respectively: 3.79(7.54) *vs* −2.08(10.79); *t*(59) = 2.49; *p* = 0.02]. No statistically significant difference was observed between the groups regarding the confidence in the accuracy of the final diagnosis of the cases or in diagnostic calibration.

**Table 1:** Mean diagnostic accuracy (initial and final diagnosis), final confidence, and final calibration as a function of experimental condition (reflection group vs control group); standard deviation into brackets

Outcome	Group		Statistics
	Control (*n* = 28)	Reflection (*n* = 33)	
Accuracy (%)			
Initial	40.48 (13.36)	45.96 (12.35)	*t*(59) = 1.66; *p* = 0.10; d = 0.42
Final	38.4 (14.6)	49.7 (12.1)	*t*(59) = 3.33; *p* = 0.002; d = 0.84
Confidence (%)	58.87 (20.10)	64.29 (13.20)	*t*(59) = 1.26; *p* = 0.21; d = 0.31
Calibration (−1 a + 1)	0.20 ± 0.19	0.15 ± 0.16	*t*(59) = 1.32; *p* = 0.19; d = 0.28

No statistically significant correlation was observed between the percentage of estimated number of right answers provided by the students at the end of the resolution of all of the cases and the students’ actual performance in both the RG (*r* = −0.05; *p* = 0.775) and the CG (r = 0.285; *p* = 0.142), suggesting that the students were unable to predict their own performance when diagnosing the studied cases.

Table 2 presents the results of the post hoc analysis with the split of the cases according to level of complexity. There was a significant main effect of case difficulty, *F*_(1, 59)_ = 192.15, *p* < 0.001, η_2_ = 0.75, with calibration showing to be worse on more difficult than on easier cases. The interaction was also significant, *F*_(1, 59)_ = 7.72, *p* = 0.007, η_2_ = 0.12, because while the two conditions performed similarly on difficult cases, the pattern observed on the easy cases differed. Whereas the CG tended towards overconfidence, the RG showed a slight tendency towards underconfidence.

**Table 2:** Relationship between experimental condition and accuracy, confidence, and calibration as a function of case difficulty

Difficulty		Group		*p*-value
	Overall	Control (*n* = 28)	Reflection (*n* = 33)	
*Easier cases*				
Accuracy (%)	69.1 ± 22.3	60.1 ± 23.3	76.8 ± 18.6	0.004
Confidence (%)	67.7 ± 17.4	65.9 ± 21.0	69.2 ± 13.7	0.473
Calibration (−1 a + 1)	−0.01 ± 0.21	0.06 ± 0.22	−0.08 ± 0.19	0.016
*More difficult cases*				
Accuracy (%)	19.9 ± 13.5	16.7 ± 12.8	22.7 ± 13.7	0.080
Confidence (%)	55.9 ± 18.6	51.8 ± 22.1	59.3 ± 14.5	0.131
Calibration (−1 a + 1)	0.36 ± 0.20	0.35 ± 0.20	0.37 ± 0.20	0.783

## Discussion

The present study aimed at investigating the effects of deliberate reflection on the accuracy of diagnosis in dermatology cases and on diagnostic calibration. The findings were only partially in line with our initial hypotheses. Students who engaged in deliberate reflection on the dermatological cases showed higher diagnostic accuracy than those who did not. The effect was substantial. Deliberate reflection, however, failed to induce significant differences in students’ confidence and diagnostic calibration.

The positive effect of deliberate reflection on diagnostic accuracy, especially when cases are complex, has been demonstrated in several previous experimental studies [20, 24]. However, this research has always been conducted in medical specialties such as internal medicine or emergency medicine, which involve recognition and integration of relevant pieces of evidence from a case, usually requiring sequential reasoning. Whether deliberate reflection would also improve diagnoses of dermatological lesions has not yet been investigated. Diagnosis in dermatology relies mostly on visual perception, depending on the clinician’s ability to recognize and classify lesions based essentially on their visual appearance, with pattern recognition playing a key role [25]. Nevertheless, our findings suggest that reflection on these visual aspects results in a better diagnostic performance. Reflecting to search for contradictory evidence apparently may help repair a wrong initial diagnosis even when recognition of relevant features has an essentially visual nature. Notice that the level of complexity of the cases, which the final diagnostic accuracy scores suggest were not straightforward for the study participants, may have left room for reflection to help. The positive effect of reflection on diagnostic accuracy is relevant for clinical practice, particularly considering the high prevalence of skin lesions, which have to be initially assessed by non-specialists such as general practitioners, with potentially serious consequences of missed diagnoses for patients [26].

Contrary to our expectation, reflecting upon the cases did not help align student confidence in the accuracy of their diagnoses and their actual accuracy. Students showed similar overconfidence when they engaged in reflection and when they did not. Studies in psychological research showed overconfidence to decrease when participants were requested to present reasons contradicting their answers, because such a request was suggested to counteract our tendency to neglect evidence that goes against our first impressions [14]. By drawing attention to findings in the case that go against one’s initial diagnosis, deliberate reflection might act in a similar way. What could then explain that it failed to improve calibration? One may argue that because diagnosing dermatology lesions relies heavily on pattern recognition, deliberate reflection could make students feel more confident as they were able to compare patterns thereby potentially increasing rather than decreasing overconfidence. The lack of difference in confidence between the two groups, however, does not seem to support this conjecture. The potential influence of deliberate reflection on judgments of confidence would decrease if people in fact do not estimate their performance solely based on their experience with the problem at hand. One might think that students judge how well they are solving a clinical case by monitoring their actual experience throughout the diagnostic process (for instance: Is it taking too long? Are they struggling to make sense of the findings?). Research has shown, however, that rather than from such ‘bottom-up’ approach, people’s estimates of their performance arise largely from a ‘top-down’ approach [27]. People start with their prior beliefs about their ability to perform a type of task and use these beliefs to judge how well they are performing in the specific task. Such a top-down approach based on preconceived judgments of competence might make it more difficult to gain from deliberate reflection. This would be particularly true for less knowledgeable participants due to the Dunning-Kruger effect [28], the well-established phenomenon showing that poor performers are ‘doubly cursed’: the lack of expertise that makes them unable to give right responses also deprives of from the ability to know when their answers are right or wrong.

The post hoc analysis distinguishing between easy and difficult cases seems in line with what would be expected from the Dunning-Kruger effect. Diagnostic calibration proved to be worse in more difficult cases, because students maintained a confidence level similar to that observed for easy cases despite the accentuated reduction in accuracy. That means, when participants were more knowledgeable on the to-be-solved problems (easy cases) calibration was much better than when they lacked enough knowledge (difficult cases). Moreover, when knowledge was available, on the easy cases reflection changed the direction of the miscalibration, eliminating the (already small) overconfidence. Reflection did not induce any significant difference in diagnostic calibration, however, on the difficult cases when participants apparently did not have enough knowledge to benefit from further studying the case to recognize evidence pointing to the correct diagnosis and realize that their initial diagnosis was wrong. This different pattern observed between easy and difficult cases in our study is also in line with the hard-easy effect, first described by Lichtenstein and Fischhoff [29], showing that the excess in participants’ confidence when answering general knowledge questions increased in direct proportion to the difficulty of the questions.

In the realm of clinical decision-making, Meyer et al. [23] also observed that both the accuracy and the confidence were less aligned when doctors had to deal with more difficult cases. The excess of confidence regarding accuracy also increased when the cases became more difficult, contrary to the initial hypothesis of these authors that both accuracy and confidence would diminish as the doctors were faced with more complicated cases. Our study adds to this previous research to reinforce the idea that diagnostic calibration seems to be a knowledge-dependent phenomenon. The same knowledge that is required to accurately diagnose a problem is also necessary to make accurate confidence judgments and even, it seems, to benefit from an aid such as deliberate reflection as a tool to improve calibration.

If this proves true, devising approaches to improving diagnostic calibration is indeed a complex, challenging endeavour. The difficulty to improve the excess of confidence is an old concern. Fischhoff [30], upon reviewing different models and attempts to eliminate this bias, revealed the lack of the effectiveness of certain strategies, such as the introduction of warnings, rewards, or the providing of clear instructions, and concluded that the excess of confidence is moderately robust to change.

The present study has some limitations. The small sample size was sufficient to demonstrate a positive effect of reflection on diagnostic accuracy, but it may not have had enough power to show an effect on diagnostic calibration, if a small one did in fact exist. The subanalyses according to the difficulty of the case must also be evaluated cautiously, given that the difficulty was not defined a priori, but rather through the students’ own performance in this study. To avoid the risk of contamination, we opted to allocate all students who were together at the time of the study in a particular rotation to the same experimental condition. We systematically alternated the allocation of rotation groups to the conditions, but there was an unexpected gender imbalance between clinical rotations resulting in a predominance of women in the reflection group. Few studies have analyzed the question of gender in relation to confidence among medical students. Blanch et al. [31] showed that, during an objective structured clinical examination, female medical students were generally less self-confident than their male counterparts. As our study showed an excess of confidence in relation to accuracy, a lower confidence among women would favour the reflection group concerning calibration, but this was not observed. Hence, we consider that the different distribution of genders among the groups did not interfere with our main results. Finally, the study was performed with final-year medical students only, and it is not known whether the findings apply to experienced clinicians.

The present study has important implications for medical education. This was the first study to demonstrate that deliberate reflection improves the diagnostic accuracy in dermatological lesions. Regarding the diagnostic calibration, our findings reinforce prior studies that show a low correlation between confidence and accuracy, which is even worse for difficult cases. They suggest that lack of knowledge affects ability to make not only accurate diagnoses but also accurate judgments of confidence and point to the complexity involved in tackling the problem of diagnostic calibration. Further research should investigate whether our findings apply to more experienced clinicians and study other interventions that may help improve calibration.
